# Evaluation of the Protective Effect of Compound Kushen Injection Against Radiation- induced Pneumonitis in Mice

**DOI:** 10.21203/rs.3.rs-3880937/v1

**Published:** 2024-01-30

**Authors:** Ting Xu, Sharmistha Chakraborty, Daoyan Wei, Megan Tran, Robyn Rhea, Bo Wei, Phuong Nguyen, Mihai Gagea, Lorenzo Cohen, Zhongxing Liao, Peiying Yang

## Abstract

Background Radiation-induced lung injury (RILI) via inflammation is a common adverse effect of thoracic radiation that negatively impacts patient quality of life and survival. Compound kushen injection (CKI), a botanical drug treatment, was examined for its ability to reduce RILI, and inflammatory responses and improve survival in mice exposed total lung irradiation (TLI). CKI’s specific mechanisms of action were also evaluated. Methods C3H mice underwent TLI and were treated with CKI (2, 4, or 8 mL/kg) intraperitoneally once a day for 8 weeks. The effects of CKI on survival were estimated by Kaplan-Meier survival analysis and compared by log-rank test. RILI damage was evaluated by histopathology and micro-computed tomography (CT). Inflammatory cytokines and cyclooxygenase metabolites were examined by IHC staining, western blot, and ELISA. Results Pre-irradiation treatment with 4 or 8 mL/kg CKI starting 2 weeks before TLI **or** concurrent treatment with 8 mL/kg CKI were associated with a significantly longer survival compared with TLI vehicle-treated group ( *P* < 0.05). Micro-CT images evaluations showed that concurrent treatment with 8 mL/kg CKI was associated with significantly lower incidence of RILI ( *P* < 0.05). Histological evaluations revealed that concurrent TLI treatment of CKI (4 and 8 mL/kg) significantly reduced lung inflammation (p < 0.05). Mechanistic investigation showed that at 72 hours after radiation, TLI plus vehicle mice had significantly elevated serum IL6, IL17A, and TGF-β levels compared with non-irradiated, age-matched normal mice; in contrast, levels of these cytokines in mice that received TLI plus CKI treatment were lower than those in the TLI plus vehicle-treated mice ( *P* < 0.05) and similar to the nonirradiated mice. IHC staining showed that the CKI treatment led to a reduction of TGF-β positive cells in the lung tissues of TLI mice (P < 0.01). The concurrent CKI with TLI treatment group had a significant reduction in COX-2 activity and COX-2 metabolites compared with the TLI vehicle-treated group ( *P* < 0.05). Conclusions These data suggest that CKI treatment was associated with reduced radiation-induced inflammation in lung tissues, reduced RILI, and improved survival. Further investigation of CKI in human clinical trials as a potential radioprotector against RILI to improve patients’ quality of life and survival is warranted.

## BACKGROUND

Radiation-induced lung injury (RILI) is common in patients with lung cancer treated with thoracic radiation and is symptomatic in 15–40% of patients (1, 2). The median time to development of symptomatic RILI is 4–6 months after completion of chemoradiation with a range from the end of radiation up to 12 months (3). RILI can cause multiple symptoms including shortness of breath, fever, chest pain, cough, hypoxia, and respiratory failure, or even death, which significantly impacts quality of life and negatively affects survival(4). At the tissue level, RILI presents as increases infiltration of inflammatory cells and fluid accumulation in alveoli, interstitial edema and epithelial degeneration followed by regeneration, intrusion of the bronchial epithelium into the alveoli, disruption of endothelial integrity and microvasculature, and atelectasis (5). Acute RILI often progresses to irreversible chronic fibrosis, resulting in decreased pulmonary function and hypoxia, which significantly impacts patients' quality of life (6). For patients with RILI that requires intervention, steroids and supplemental oxygen are the only reliable treatment because there is no effective drug that can specifically mitigate RILI. Amifostine is among the most extensively investigated thiols for protecting normal tissue from various cancer therapies. However, its clinical use in RILI prevention has not gained popularity owing to its high incidence of side effects (7) and high heterogeneity of therapeutic effect in clinical trials (8). The fact that no effective drug therapy is available for RILI represents a significant unmet clinical need.

The progressive dysregulation of lung tissue caused by RILI can begin immediately after radiation exposure through the production of free radicals with increased oxidative stress and associated inflammatory cytokine response (9). Two primary mechanisms trigger radiation-induced tissue injuries: DNA double-strand breaks and the generation of reactive oxygen species (10, 11). In addition, radiation-induced damage to DNA or cytoplasmic organelles activates intracellular signaling that leads to altered gene expression (12). Many cytokines and chemokines are elevated in both the serum and bronchoalveolar fluid of humans and mice with RILI (13, 14). Among these proteins, elevated interleukin (IL) 6 levels are predictive for RILI (15, 16). Transforming growth factor β (TGF-β), derived from inflammatory cells and, to a lesser degree, from pneumocytes and fibroblasts, is a key cytokine involved in the fibrotic process (6, 17, 18). More recently, IL17A was identified as an emerging target for RILI treatment (19).

Compound kushen injection (CKI) is a hot water extract from the herbs kushen (*Sophora flavescentis radix*) and baituling (*Heterosmilax yunnanensis rhizoma*). Kushen has long been used to treat tumors, inflammation, and other diseases, such as viral hepatitis, enteritis, viral myocarditis, arrhythmia, and skin diseases (e.g., colpitis, psoriasis, eczema) in China (20). The major bioactive components of kushen are matrine and oxymatrine. CKI is manufactured in a GMP facility and consists of over 200 different chemical components, predominantly alkaloids and flavonoids, which are mainly derived from kushen (21).

As a Chinese Food and Drug Administration-approved product, CKI has been used regularly in oncology clinics in China for more than 20 years (20, 22, 23). It has been used in combination with chemotherapy for the treatment of gastric, liver, and non-small cell lung carcinomas (24) and has also been observed to alleviate the toxicity of radiochemotherapy (25). Treatment regimens include 12–30 mL intravenous injection daily for 4–8 weeks and provided concurrently in patients getting radiotherapy (20, 22, 23). In a systematic review and meta-analysis of 13 clinical studies, Wang et al. (22) reported that CKI significantly reduced radiation-induced adverse events, including radiation pneumonitis, esophagitis, and bone marrow suppression, and improved quality of life in non–small cell lung cancer patients. Most recently, the effectiveness of CKI in reducing the incidence of grade ≥ 2 RILI and symptom burden and improving patients' quality of life in patients with lung cancer was documented (26). A limitation is that all the prior clinical research was conducted in China.

Preclinical research of CKI shows that CKI treatment leads to the cell cycle arrest and autophagic cell death by upregulation of LC3-I and II protein expression in human NSCLC H1975 and H1650 cells and sensitizing gefitinib by down regulation of PI3K/mTOR pathways (27, 28). The effect of CKI on apoptosis was also reported in breast cancer MCF-7 and liver cancer HepG2 cells (29). CKI inhibits sarcoma growth and tumor-induced hyperalgesia via TRPV1 signaling pathways and suppresses human breast cancer stem-like cells by downregulating the canonical Wnt/β-catenin pathway (30). However, CKI's mechanisms regarding its effects on RILI are still under investigation representing a significant knowledge gap.

Thus, the goal of the present study was to evaluate the impact of CKI treatment on RILI and identify its mechanism of action in mouse models. We hypothesized that CKI reduces RILI via its anti-inflammatory effect and that it can potentially treat RILI in patients in North America.

### METHODS

All procedures were performed according to the relevant guidelines, rules, and regulations of The University of Texas MD Anderson Cancer Center.

## Study drug

CKI (batch #20180301 or #20200110) was provided by Shanxi Zhendong Pharmaceutical Co. (Changzhi, Shanxi, China). It was prepared through the percolation method as specified in Appendix 10 of the Chinese Pharmacopoeia 2010 Edition. The detailed preparation procedure is described in the supplementary material (see supplement). Chemical fingerprint for CKI and the quantification of major chemical composition using High Performance Liquid Chromatography (HPLC) were reported previously (31). Figure S1 shows the quantification and consistency of matrine and oxymatrine in three batches of CKI, two of which were used in this study, using Liquid Chromatography with tandem mass spectrometry (LC/MS/MS). Total volumes of 200 μL solutions were prepared for each injection with CKI and saline. The amount of CKI included in each injection at doses of 2, 4 and 8 mL/kg were 50, 100 and 200 μL CKI diluted with 150, 100 and 200 μL saline.

## Other materials

Prostaglandin $E_2$ ($PGE_2$), $PGD_2$, and its relevant internal standards $PGE_2$-d4 and $PGD_2$-d4 as well as COX-2 activity assay kit (#760151) were purchased from Cayman Chemical (Ann Arbor, MI). Anti-Cyclooxygenase-2 (Anti-COX-2) and anti-15-PGDH antibodies were obtained from Novus Biological (Centennial, CO).

## Animals

The research protocol was approved by the Institutional Animal Care and Use Committee of The University of Texas MD Anderson Cancer Center. Female C3H/KamL mice from the Department of Experimental Radiation Oncology, MD Anderson and female C3H/heN mice (Charles River, Wilmington, MA) were used in this study. As described previously, the C3H mouse strain is well characterized in studies of RILI intervention (32, 33). The study mice were 12 to 16 weeks old and maintained in accordance with the guidelines of our Office of Research Administration and Institutional Animal Care and Use Committee. They were housed at the institutional animal facility under controlled temperature and humidity levels and a 12:12 h light-dark schedule. They were given lab chow diet (Harlan Laboratories, Indianapolis, IN) and water ad libitum.

## Radiation procedure

Total lung irradiation (TLI) was performed using an XRAD 225Cx (Precision X-Ray, North Branford, CT). Irradiation was performed according to the standard operating procedure of our institution’s Small Animal Imaging Facility. The setup for TLI consisted of a 225 kVp, 13.3 mA beam with a 0.15 mm copper filter and 20 mm × 20 mm beam collimator. Radiation was delivered at a dose rate of 300 Gy/min from the anterior–posterior/posterior–anterior direction using two parallel opposing beams. Mice were anesthetized with inhaled anesthesia (5% isoflurane for induction and 1.5%-3% for maintenance) and positioned so that only the thorax was exposed in the radiation field.

## Experiments

**Experiment 1: Determine the optimal timing and dose response of CKI treatment after single dose radiation on survival.**

A single radiation dose of 13.5 Gy was used to determine the efficacy of CKI in preventing RILI and its relevant mechanism of action, as a previous study showed that C3H mice developed pneumonitis after they were irradiated at this dose level (32, 34, 35). The optimal timing of CKI delivery was investigated using three different schedules: 1) starting 2 weeks prior to TLI; 2) starting concurrently on the same day as TLI; or 3) starting 9 weeks after TLI, which is the median time for the development of acute RILI (see treatment schema in Fig. 1A). When CKI and TLI were given on the same day, mice were irradiated before receiving CKI treatment.

CKI was administered to mice (n = 16 to 20 per dose group) once per day five days/week via intraperitoneal injection at doses of 2, 4, or 8 mL/kg for a total of 8 weeks (40 injections), with a 1-week break after the initial 4 weeks. The treatment schedule was based on the CKI administration schedule routinely used in oncology clinics in China. The 4 mL/kg dose was equivalent to a clinical dose of 20 mL/day, which is the dose approved by the China Food and Drug Administration for this product (22, 23).

Blood was collected from the facial vein at 2, 4, 8 weeks after TLI. Micro-computed tomography (CT) imaging was performed 1 week before and 8–12 weeks after TLI. Lung tissues were collected and processed for histopathological evaluation at the termination of the study. Survival and RILI were evaluated and compared between the treatment groups to determine the optimal timing and dose of CKI.

**Experiment 2: Determine the effect of CKI on survival at different radiation dose levels.**

Mice were randomly assigned to TLI only and TLI + CKI groups. Mice were irradiated at a range of single radiation doses (11.75-14 Gy, n = 3 to 9 per group) to establish radiation dose response on survival. In the TLI + CKI group, CKI 8mL/kg was injected to mice concomitantly with TLI and then daily for 8 weeks, as this was the most effective dose and schedule suggested from the results of experiment 1.

## Experiment 3: Effect of CKI on the mediators of RILI

To investigate the potential mechanism(s) for the effects of CKI at reducing RILI, mice (n = 3–8 per group) were treated with 13.5 Gy TLI and CKI at doses of 4 and 8mL/kg concomitantly and then daily for 8 weeks of CKI treatment. Blood was drawn from the facial vein at 72 h for cytokine analysis. Micro-CT imaging and lung tissue collections were performed at 2, 4, 6 weeks after TLI for RILI evaluation. In addition, lung tissues were processed as formalin fixative or flash frozen for related experiments.

## Endpoints evaluations

## Survival evaluation

Survival is a common primary endpoint for the assessment of the radioprotective effects of a defined agent in C3H mice (33). Mice were weighed once per week and observed daily for signs of morbidity (hunched posture, progressive weight loss of more than 20%, labored breathing). Mice were euthanized by $CO_2$ inhalation when they lost 20% of body weight or became moribund.

## RILI evaluation

## Histopathology evaluation

Formalin-fixed lung tissues were embedded in paraffin, and sections were stained with hematoxylin and eosin (H&E) for histopathological examination. The RILI score was established based on the evaluation of H&E-stained lung sections as follows: grade 0, normal tissue; grade 1, minimal tissue changes affecting 1–10% of the tissue; grade 2, mild tissue changes affecting 11–20% of the tissue; grade 3, moderate tissue changes affecting 21–40% of the tissue; and grade 4, marked tissue changes affecting 41–100% of the tissue. A certified veterinary pathologist blinded to experimental groups performed histopathological examinations of 4–5 lung tissues from each lobe.

## Imaging evaluation

Micro-CT imaging was performed using XRAD 225Cx (Precision X-Ray) within 1 week before radiation and again 2–12 weeks after radiation. Briefly, the mice were anesthetized with inhaled anesthesia (5% isoflurane for induction and 1.5%-3% for maintenance). When the mice were fully anesthetized, a 22-gauge endotracheal tube was placed using a BioLite mouse intubation system (Braintree Scientific, Braintree, MA). The CT parameters used were 60 kV, 4 mA, and 3 rpm. The mice were mechanically ventilated at 60 breaths per minute throughout the procedure, and a 20 second breath hold was applied during image acquisition at 20 cm$H_2O$. The pressure was monitored through an inline manometer. After image acquisition was complete, the mice were extubated and recovered in a clean and warm cage. Two to three certified radiation oncologists blinded to experiment groups reviewed the Micro-CT images for RILI evaluation.

## Immunohistochemistry

Formalin-fixed lung tissues were embedded in paraffin, and sections were stained with COX-2 (Cell Signaling, #12282), TGF-β (Abcam, Waltham, MA), or β-catenin (Cell Signaling, #8480) antibodies for immunohistochemistry (IHC) analyses. For the IHC staining, 4-μm sections were stained following the standard operating procedure of our institution's histopathological core facility. The stained slides were scanned with an Aperio AT2 bright-field slide scanner (Durham, NC), and IHC staining was quantified with Aperio image analysis algorithms for each lobe of the lung tissues (36).

## Serum cytokine analysis

Given that proinflammatory cytokines, including IL6, IL17, and TGF-β, have been associated with RILI and that changes in these cytokines appeared soon after radiation in C3H mice (13, 37, 38), the serum levels of these cytokines were determined by enzyme-linked immunosorbent assay (ELISA) kits (R&D Systems, Minneapolis, MN, and Invitrogen/Thermo Fisher Scientific, Waltham, MA).

## COX-2 activity in lung tissue

Lung tissues were collected after perfusion with Tris pH 7.4 and homogenized with cold buffer containing 0.1M Tris pH 7.8, 1mM EDTA (10 ml/g of tissue). The COX-2 activity was assessed according to the manufacture instruction. Briefly, the lung tissue supernatants were collected and used for the assay. An aliquot of 40 μl lung tissue supernatant was mixed with 10 μl of Hemin, 20 μl of Colorimetric substrate, and 20 μl of arachidonic acid in 110 μl of assay buffer. The absorbance was read at 590 nM. COX-2 activity was calculated by subtracting average background sample and from its corresponding sample or inhibitor treated sample. The COX-2 activity was calculated per sample using TMPD extinction coefficient of 0.00826 $mM^{-1}$ following instructions from the manufacturer. (Cayman Chemical, Cat no 760151).

## Eicosanoid-profiling analysis

We and others have demonstrated the importance of COX-2–mediated inflammatory mediators in RILI (32, 39), thus, we further determined the levels of cyclooxygenase metabolites in lung tissue using LC/MS/MS, as described previously (40). Briefly, analyses were performed using an Agilent 6460 Triple Quad mass spectrometer (Santa Clara, CA) equipped with an Agilent 1200 HPLC system. Eicosanoids were separated using a Kinetex C18 2.6-µm, 2.0 × 100-mm column (Phenomenex, Torrance, CA). The mobile phases used 0.1% formic acid in water (phase A) and 0.1% formic acid in acetonitrile (phase B). For the analyses of prostaglandins $PGE_2$ and $PGD_2$, separation was achieved using a linear gradient of 20%-98% of 0.1% formic acid in acetonitrile (phase B) with a total time of 33 min. The flow rate was 400 µL/min with a column temperature of 30°C. The sample injection volume was 15 µL. Samples were kept at 4°C during the analysis. The mass spectrometer was operated in the electrospray negative-ion mode with a gas temperature of 350°C, a gas flow rate of 10 L/min, and a nebulizer pressure of 20 psi. The temperature of the sheath gas was 350°C and the sheath gas flow rate was 12 L/min. The capillary voltage was − 2900 V. Fragmentation was performed for all compounds, using nitrogen as the collision gas. COX-2 metabolites were detected using electrospray negative ionization and multiple-reaction monitoring of the transitions at mass-to-charge ratios of 351.2 →271.2 for $PGE_2$/$PGD_2$ and 355.2 →275.2 for $PGE_2$-d4/$PGD_2$-d4. The results were expressed as nanograms of prostaglandins per milligram of protein.

## Immunoblotting

Frozen lung tissue samples were washed with cold PBS and chopped in lysis buffer containing proteinase and phosphatase inhibitor cocktails. They were then transferred to vials containing silica beads and homogenized in Precellys homogenizer (Bertin Technologies). The tissue lysates were collected after being centrifuged at 16,000g in 4 °C. The immunoblotting was performed using the automated western blotting system. The Jess™ Simple Western system (ProteinSimple, San Jose CA, USA,) is a system that automatically separates and immunodetects proteins by size through its capillaries. To quantify the interested protein expression of treated tissues, the standard method for 12-230-kDa Jess separation module (SM-W004) was followed. The final protein concentration of tissue lysate (0.4 to 1 mg/mL) was obtained by mixing measured tissue lysate (0.6 to 2 mg/mL) with 0.1X Sample buffer and Fluorescent 5X Master mix (ProteinSimple). This mixture was denatured at 95°C for 5 min. Primary antibodies of interest (COX-2 and 15-PGDH) and tubulin were prepared by mixing antibody diluent with ratio from 1:10 to 1:50. Secondary antibodies were prepared by combining chemiluminescence antibody and fluorescence antibody. After that, tissue lysate samples were loaded into the Ladder (12-230-kDa) plate, followed by the loading of antibody diluent, primary antibody, secondary antibody, luminol-s and peroxide mixture (for chemiluminescence detection), and wash buffer according to the manufacturer instruction. Digital images of chemiluminescence and fluorescence of the interested proteins were captured with Compass Simple Western software (version 4.1.0, Protein Simple), and the area was used for the quantification of the proteins.

## Statistical analyses

Kaplan-Meier survival functions and log-rank tests were performed to compare survival durations between the treatment groups. RILI incidence rates were compared between the control and CKI-treatment groups using the Fisher’s exact test. Differences between the groups’ means for other outcomes were determined using the student *t*-test or one-way ANOVA. The statistical significance threshold was $P < 0.05$.

## RESULTS

### CKI treatment was associated with improved survival of C3H mice after TLI

The mice that received pre-irradiation CKI starting 2 weeks before TLI had a significantly longer median survival compared with the mice in the TLI vehicle (saline) control group, with a difference of 15 days at 4 mL/kg (median ± standard error [SE]: 101.0 ± 5.0 days vs. 86.0 ± 0.8 days, $P = 0.004$) and a difference of 8 days at 8 mL/kg (median ± SE: 94.0 ± 7.6 days vs. 86.0 ± 0.8 days, $P = 0.025$; Fig. 1B). Similarly, the mice that received concurrent 8 mL/kg CKI starting at the same day as TLI had longer median survival compared with the TLI vehicle control group, with a difference of 16 days (median ± SE: 108.0 ± 0.4 days vs. 92.0 ± 8.4 days, $P = 0.025$ Fig. 1C). However, mice that received concurrent 4 mL/kg CKI starting at the same day as TLI did not survive significantly longer than the control group (median ± SE: 101.0 ± 6.4 vs. 92.0 ± 8.4 days, $P = 0.126$; Fig. 1C). There were no significant differences in survival between 2 mL/kg CKI treatment groups and the control group regardless of when the CKI was administered (Fig. 1B**&C, S2**). Additionally, there were no statistically significant differences in the median survival durations of the mice treated with CKI at 9 weeks after TLI (**Figure S2**). The weight changes of the study mice after TLI are plotted in **Figure S3**. Mice weight significantly decreased in the first 2 weeks after TLI and started to recover after 2 weeks. Weight changes did not significantly differ over time among groups.

To further understand whether the protective effect of CKI against RILI depends on the given dose of radiation, we conducted a CKI effect study in mice that received concurrent TLI + vehicle or TLI + 8 mL/kg CKI with radiation doses of 11.75, 12.5, 13.25, or 14 Gy. In vehicle control mice, mice that received radiation 14 Gy had significantly shorter median survival (median ± SE: 86.0 ± 1.8 days, $P < 0.05$; Fig. 2A) than the mice that received 11.75 or 12.5 Gy (median ± SE: 94.0 ± 0.8 and 98 ± 5.7 days respectively). Other three lower radiation doses, 11.75, 12.5, and 13.25 Gy, didn't show different effects in survival (median survival time: 92 to 98 days). We found that concurrent 8 mL/kg CKI treatment significantly prolonged survival in all radiation dose levels with median survival prolonged by 13–25 days ($P < 0.05$, Fig. 2B). These results suggest that CKI exerted protection against RILI-associated mouse death within a defined dose of radiation from 11.75 to 14 Gy which induced different extents of RILI.

#### CKI reduced the incidence of RILI in C3H mice.

Micro-CT imaging of lung tissues was performed before the start of treatment and 1–3 months after 13.5 Gy TLI to monitor RILI in mice treated with vehicle control and CKI at 4 mL/kg and 8 mL/kg started concurrently. Micro-CT images showed representative images of lung tissues of mice in the normal, non-irradiated condition (Fig. 3**Aa**), TLI + vehicle with subsequent RILI (Fig. 3**Ab**), and TLI + CKI 8 mL/kg with subsequent RILI (Fig. 3**Ac**), as well as of healthy lung tissues in TLI + CKI 8 mL/kg treated mice (Fig. 3**Ad**). Both 4 mL/kg (n = 8) and 8 mL/kg (n = 13) CKI treatment groups had lower incidences of RILI than that of the vehicle control group (n = 5) with RILI rates of 25% (4 mL/kg) and 8% (8 mL/kg) vs. 80% (vehicle) (Fig. 3B).The difference between the 4 mL/kg CKI and the vehicle was not statistically significant ($P =$

0.086); however, the 8 mL/kg CKI significantly reduced the incidence of RILI compared to the vehicle ($P$ < 0.008), suggesting that CKI 8 mL/kg can slow the development of RILI in C3H mice.

To further understand the pathological changes and group differences that occurred in TLI + vehicle vs. TLI + CKI groups, a certified veterinary pathologist blinded for experiment groups performed histopathological examinations of the formalin-fixed lung tissues. Outcomes revealed that the TLI + vehicle control group was more likely to experience acute interstitial pneumonia, as evidenced by acute, diffuse, alveolar, and neutrophilic inflammation with perivascular edema and intra-alveolar proteinaceous exudation with formation of hyaline membranes (Fig. 4**Aa**). Consistent with previous findings, no fibrosis was noted in these mice (41). H&E staining of the lung tissues derived from TLI + CKI (4 and 8 mL/kg)-treated mice showed that these animals had a much lower degree of RILI, as evidenced by their healthy and dense alveolar networks (Figs. 4**Ab-c**). Quantifications of the histopathological staining (Fig. 4B) showed statistically significantly reduced RILI in CKI-treated (4 and 8 mL/kg) mice compared with vehicle-treated mice, suggesting that CKI substantially prolonged the survival of the irradiated C3H mice by reducing RILI.

## CKI inhibited inflammatory cytokines in C3H mice exposed to TLI

To understand the potential mechanisms of action associated with CKI's preventive effects against RILI in C3H mice, proinflammatory cytokines were measured in the serum of nonirradiated control mice and at 72 hours after irradiation in mice treated with 13.5 Gy TLI + vehicle started concurrently, and mice treated with 13.5 Gy TLI + CKI (4 and 8 mL/kg) started concurrently (n = 3–4 mice per group). IL6 levels in the TLI + vehicle group (4.75 ± 0.59 pg/mL) were 8.0-fold higher than in the nonirradiated control mice (0.58 ± 0.38 pg/mL) (Fig. 5A). TLI + CKI (4 and 8 mL/kg CKI) significantly reduced the mean IL6 level to 1.09 ± 0.15 pg/mL and 0.70 ± 0.29 pg/mL ($P$ < 0.001; Fig. 5A), respectively, which was comparable to that of the nonirradiated control mice. Similarly, the IL17A levels in TLI + CKI (4 and 8 mL/kg)-treated mice were 73% lower than those in TLI + vehicle control mice ($P$ < 0.01; Fig. 5B). Furthermore, the TGF-β levels in TLI + CKI (4 and 8 mL/kg)-treated mice were 24.49 ± 2.63 ng/mL and 16.61 ± 8.60 ng/mL, respectively, which were also significantly lower than that of TLI + vehicle control mice (42.39 ± 3.80 ng/mL, $P$ < 0.05; Fig. 5C). The reduction in radiation-induced TGF-β by CKI was dose dependent. Again, the TGF-β level in mice treated with TLI + 8 mL/kg CKI was similar to that of nonirradiated control mice (16.36 ± 1.23 ng/mL). Furthermore, to investigate the temporal change of cytokines in TLI mice, serum IL6 was measured at 2, 4 and 8 weeks after TLI and expression of TGF-β in lung tissues was measured at 2 weeks after TLI. Levels of serum IL6 in TLI control group were 2.5-fold higher ($P$ < 0.01) than that of non-radiation control mice at 2 weeks after TLI but recovered at 4 and 8 weeks. Mice treated with CKI 4 or 8 mL/kg showed stable low levels of IL6 as non-radiation control at 2, 4, and 8 weeks after TLI (Fig. 5D). Consistently, IHC staining in lung tissues showed that the percent of TGF-β positive cells in TLI only mice (26.9%) were much higher than that of non-radiation control (0.9%, $P$ < 0.0035) and CKI (8mL/kg) treated mice (2.5%, $P$ < 0.0052) at

2 weeks after TLI (Fig. 5E**&F**). These data suggest that CKI markedly decreased radiation-induced inflammatory cytokine levels and exerted anti-inflammatory activity as an early response to radiation.

## CKI modulated COX-2 pathways in C3H mice exposed to TLI

COX-2 metabolites and COX-2 activity were assessed in the lung tissues of mice 4 weeks after radiation in non-irradiated, 13.5 Gy TLI + vehicle and TLI + CKI 4 and 8 mL/kg groups. As shown in Fig. 6A, there were significantly lower levels of the intralung COX-2 metabolites $PGD_2$ and $PGE_2$ 4 weeks after radiation in the CKI-treated (4 and 8 mL/kg) mice compared to the TLI + vehicle control mice ($P < 0.05$). To understand whether this reduction of COX-2 metabolites was mediated by suppression of COX-2 activity or protein expression, COX-2 activity and protein expression were measured by ELISA and IHC staining in irradiated lung tissues of the mice treated with TLI + vehicle and TLI + CKI (4 and 8 mL/kg). The COX-2 activity was significantly reduced in the lung tissues of CKI treated TLI mice compared to that of TLI + vehicle control group ($p < 0.05$). In contrast, the percentages of COX-2–positive cells in the irradiated lung tissues, treated either with vehicle or with CKI 8 mL/kg, were similar (**Figure S4A&B**). Furthermore, COX-2 protein expressions in lung tissue in TLI + vehicle and TLI + CKI (4 and 8 mL/kg) by immunoblotting were not significantly different (**Figure S4C**), suggesting that the reduction in COX-2 metabolites by CKI was not due to inhibition of COX-2 protein expression. To further understand whether CKI also affects the degradation pathway of prostaglandins, the protein level of 15-PGDH, which is the common degradation enzyme of $PGE_2$ and $PGD_2$, was examined in the lung tissues of C3H mice. CKI (8 mL/kg) treatment significantly upregulated the expression of 15-PGDH in TLI mice compared to that of TLI + vehicle group ($P < 0.05$) **(**Fig. 6C**)**, suggesting that CKI elicited reduction of $PGE_2$ and $PGD_2$ in TLI mice could be at least partially due to inhibition of COX-2 activity and the increase in the degradation of these metabolites in lung tissues.

## DISCUSSION

In the present study, we provided several lines of evidence to suggest that CKI reduces inflammation and alleviates radiation-induced pneumonitis. First, pre-irradiation or concurrent TLI and CKI 8 mL/kg was associated with improved survival of irradiated C3H mice. Second, CKI was associated with reduced incidence and severity of RILI evaluated by imaging and histopathology. To our knowledge, this preclinical study, using micro-CT imaging and histopathological results, is the first to demonstrate RILI prevention effect of CKI. Third, CKI treatment significantly reduced key proinflammatory cytokines including IL6, IL17, and TGF-β as well as activity of COX-2 supporting anti-inflammation as the hypothesized mechanism of action of CKI.

The inflammatory responses in RILI are directly associated with ongoing primary tissue damage (ref). RILI is well known to alter the levels of inflammatory cytokines such as TGF-β (18). In addition, higher plasma levels of TGF-β1, IL1α, and IL6 predict a higher risk for RILI (19, 42, 43), emphasizing the important role of activation of these cytokines in RILI. More recent studies have indicated that targeting and downregulating IL17A could be a key approach to manage various inflammatory disease models as this reduces inflammation and fibrosis (44). Furthermore, the severity of lung injury caused by radiation in mice is significantly decreased in IL17A-knockout mice (45). Interestingly, anti-inflammatory drugs such as dexamethasone and an anti-IL17A antibody reduced the concentrations of IL17A (19, 45, 46), TGF-β, and IL6 and alleviated RILI and subsequent fibrosis in mice, suggesting the importance of IL17A in inflammation and fibrosis (46). The current data examining the use of CKI demonstrated that CKI significantly reduced radiation-induced elevation of IL6, IL17A, and TGF-β in the serum of C3H mice 72 h after irradiation. The findings also revealed that the levels of TGF-β in the lung tissues of TLI mice treated with CKI at 2 weeks after irradiation were significantly reduced. A study has shown that the levels of the circulating proinflammatory cytokines such as IL6 in serum and BAL were correlated with tissues levels of the proinflammatory markers (37). Our data showed that CKI treatment not only reduced circulating levels (serum) of TGF-β, but also in the lung tissues which is consistent with a recently published mechanism study for CKI protection effect against liver fibrosis and hepatocarcinogenesis (47). This further indicates that CKI exerts its protective effect against RILI through targeting these key inflammatory cytokines in the circulation and within the lung tissue itself. However, which of the three inflammatory cytokines, or perhaps others, are the key regulatory factors that directly impact the CKI-elicited protective effects in RILI has not been evaluated in the current study and deserves further investigation. As the inflammatory response is complex and multidimensional, it is unlikely that there is one specific component driving the effects.

One of the predominant histopathological and physiological events in RILI is local inflammation. Selective COX-2 inhibitors, such as celecoxib, have been shown to significantly increase the survival of the irradiated mice when treatment started 9 weeks after radiation (32), suggesting the importance of COX-2 pathway in protecting radiation induced RILI. Prostaglandins, known inflammatory mediators, are synthesized from arachidonic acid via the actions of cyclooxygenase (COX-1 or COX-2) enzymes and degraded by the key enzyme 15-hydroxyprostaglanin dehydrogenase (15-PGDH) (48, 49). Thus, the levels of prostaglandins, especially $PGE_2$, are regulated by the COX-2 and 15-PGDH enzymes in tissues, including lung tissue. The current study found that CKI-treated mice had a decrease in COX-2 metabolites in lung tissue, including $PGD_2$ and $PGE_2$, suggesting that CKI may reduce RILI by targeting COX-2 pathways. Indeed, CKI also reduced the activity of COX-2.

A prior study found that Clarithromycin prevented RILI by downregulating COX-2 protein expression in irradiated C57B/L6 mice (50). In our study, the result of IHC staining and western blot analysis showed that CKI treatment did not reduce radiation-induced COX-2 expression, but rather slightly increased, suggesting that CKI inhibits the activity of COX-2 which then led to increased COX-2 protein expression via feedback mechanism as we have showed in a previous study (51). CKI treatment also led to upregulation of 15-PGDH expression which could at least in part contribute to the CKI-elicited degradation of prostaglandins such as $PGE_2$ and $PGD_2$. Given research has shown that 15-PGDH is abundant in both normal mouse and human lung tissues and 15-PGDH has been demonstrated as a tumor suppressor in lung and colon cancer, it would be interesting to further understand how CKI is able to upregulate 15-PGDH levels in TLI mice and reduce overall RILI. Additionally, the COX-2 metabolic pathway can be regulated by both TGF-β and NF-kB in RILI (52, 53). Examination of NF-kB in the CKI treated TLI mice might help further understand the mechanism of action of CKI in RILI.

One of the limitations of the current series of experiments is not knowing which of the purported active components of CKI exerts the protective effects for RILI. Preclinical studies have demonstrated that matrine, one of the active ingredient in CKI, significantly improves the survival of mice with lipopolysaccharide-induced acute lung injuries and alleviates pulmonary edema by inhibiting inflammatory cytokines and mediators such as tumor necrosis factor α, IL6, and high mobility group protein B1 (28), and downregulating inflammatory genes such as *IL6*, *CCL1*, and *COX2* (54). Oxymatrine, another major component of CKI, has anti-inflammatory activity; however, studies have produced mixed results regarding its role in inhibiting cytokine levels and cytokine-cytokine receptor interactions in cancer cells (55, 56). The flavonoids from kushen roots have been reported to significantly inhibit IL6, COX-2, and nitric oxide synthase in lipopolysaccharide-treated RAW 264.7 mouse macrophage cells and to suppress inflammation in a mouse arthritis model (57). However, which bioactive components (i.e., alkaloids, flavonoids) in CKI are responsible for CKI's protective effects against RILI remain unknown. Even though one can speculate that CKI's role in preventing RILI may be due to both alkaloids and flavonoids, further studies are needed to explore the specific components or combination that leads to CKI-induced reduction of RILI.

Through micro-CT imaging and histopathological tests, the current study observed that CKI treatment led to significantly less RILI, which may have contributed to the longer survival of the CKI-treated mice. However, the mice with minor or no RILI only had moderately longer survival than that of mice with RILI, suggesting that radiation-induced damage to other organs, such as the esophagus and heart, may contribute to the mortality of mice without RILI. H&E-staining of heart tissue derived from irradiated mice with or without CKI treatment did not find any significant pathological changes in these tissues (data not shown). Other radiation-induced damage, such as thrombosis and bone marrow damage, in TLI mice should be further investigated.

## CONCLUSIONS

Collectively, the current findings suggest that CKI administered on the same day as a single TLI dose and then daily for 8 weeks, with a week break, improved survival and reduced the development of RILI. CKI treatment led to decreased expression of inflammatory cytokines such as TGF-β, IL6, and IL17 and reduced activity and metabolite of intralung COX-2. These findings were supported by histopathological findings and micro-CT data showing improvements in lung structure. Further clinical studies of CKI as a potential candidate for preventing RILI are warranted.

## Abbreviations

CKI
compound kushen injection
COX-2
cyclooxygenase 2
H&E
hematoxylin and eosin
IHC
immunohistochemistry
IL
interleukin
PG
prostaglandin
RILI
radiation-induced lung injury
TGF-β
transforming growth factorβ
TLI
total lung irradiation
TNF-α
tumor necrosis factor alpha.

## Declarations

#### Ethical approval and consent to participate

Not applicable

#### Consent for publication

All authors have given their approval for publication of this paper in Radiation Oncology.

#### Availability of supporting data

Upon request, data can be obtained from the corresponding author, Dr. Peiying Yang.

#### Competing interests/authors' contributions

The authors declare that the research was conducted in the absence of any commercial or financial relationships that could be construed as potential conflicts of interest. P.Y., T.X., and Z.X.L. developed the main conceptual ideas and provided input into the study design. M.T., S.C., B.W., R.R., and T.X. performed the experiments and numerical calculations for the planned experiments. M.G. evaluated the histologic slides and facilitated data interpretation. S.C., D. W., P.Y., T.X., and Z.X.L. drafted and/or reviewed the manuscript, P.Y. and T.X. final approved the version to be submitted.

### Funding

This study was supported by Shanxi Zhendong Pharmaceutical Inc (to P. Yang). The study's micro-CT imaging was also supported by the National Institutes of Health/National Cancer Institute under award number P30CA016672 and used the Small Animal Imaging Facility.

## Figures

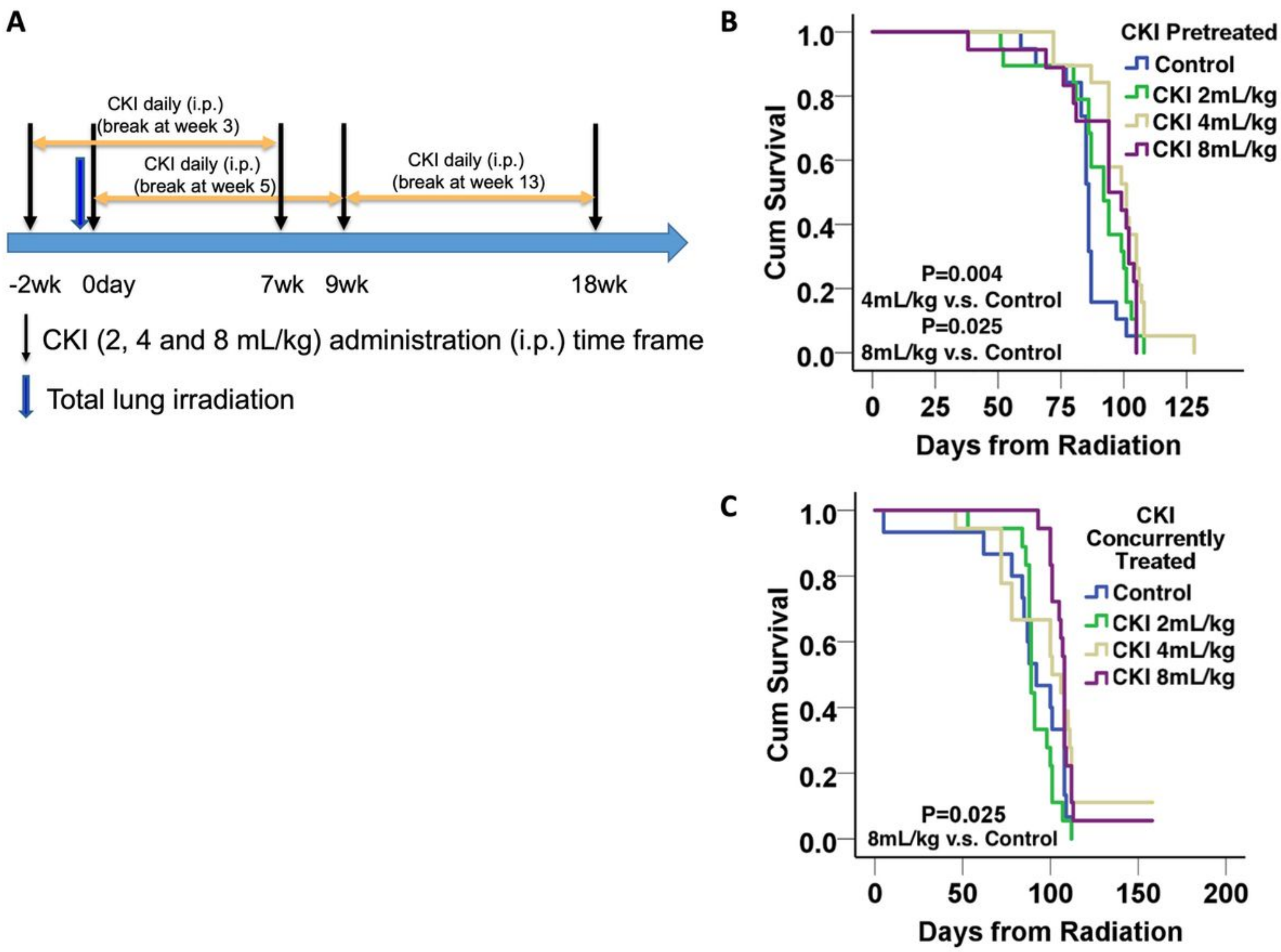

Figure 1

**Treatment schema and Kaplan-Meier survival analysis of C3H mice treated with compound kushen injection (CKI).** (A). Illustration of the treatment delivery schedules. Two cycles (5 days a week for 4 weeks/cycle), separated by a week, of CKI were given to the study mice through intraperitoneal injection. Three different administration schedules of CKI were tested, including starting CKI 2 weeks before total lung irradiation (TLI), concomitantly with TLI, and waiting until 9 weeks after TLI to start CKI. The total lung of C3H mice were exposed to a single 13.5 Gy dose and treated with the indicated doses of CKI. (B) Survival curves for C3H mice treated with CKI before irradiation (4 mL/kg CKI vs. control, *P* = 0.004; 8 mL/kg CKI vs. control, *P* = 0.025). (C) Survival curves for irradiated mice treated with CKI starting at the time of irradiation (8 mL/kg CKI vs. control, *P*= 0.025).

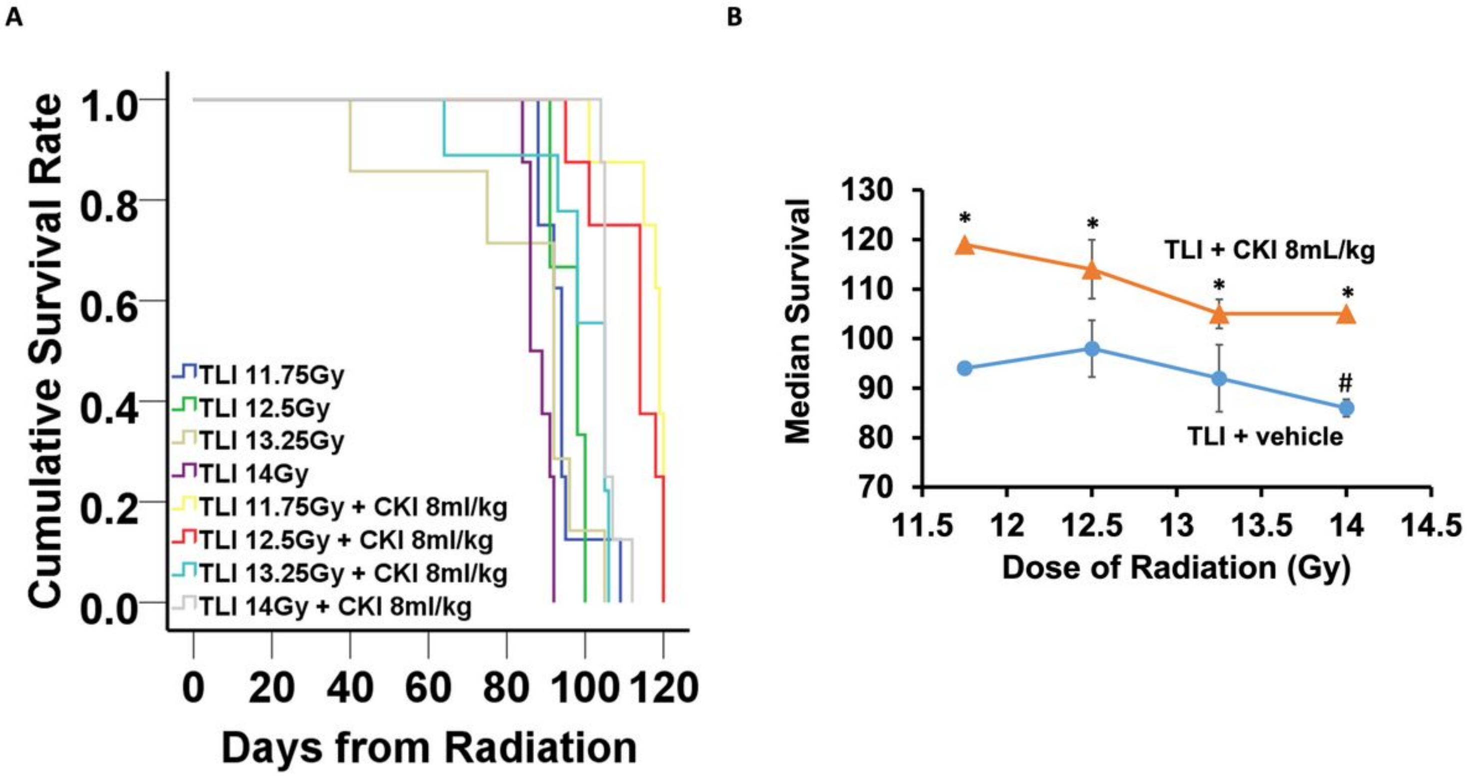

Figure 2

**Dose-dependent overall survival of C3H mice treated with CKI and TLI**. (A) Kaplan-Meier curves show statistically significant differences between median survival times in the mice that received the highest radiation dose (14 Gy) vs. lower radiation doses (# $P < 0.05$). (B) CKI treatment (8 mL/kg) significantly prolonged the median survival time of mice treated with TLI plus CKI compared to those treated with TLI and vehicle at all radiation dose levels (*$P < 0.05$).

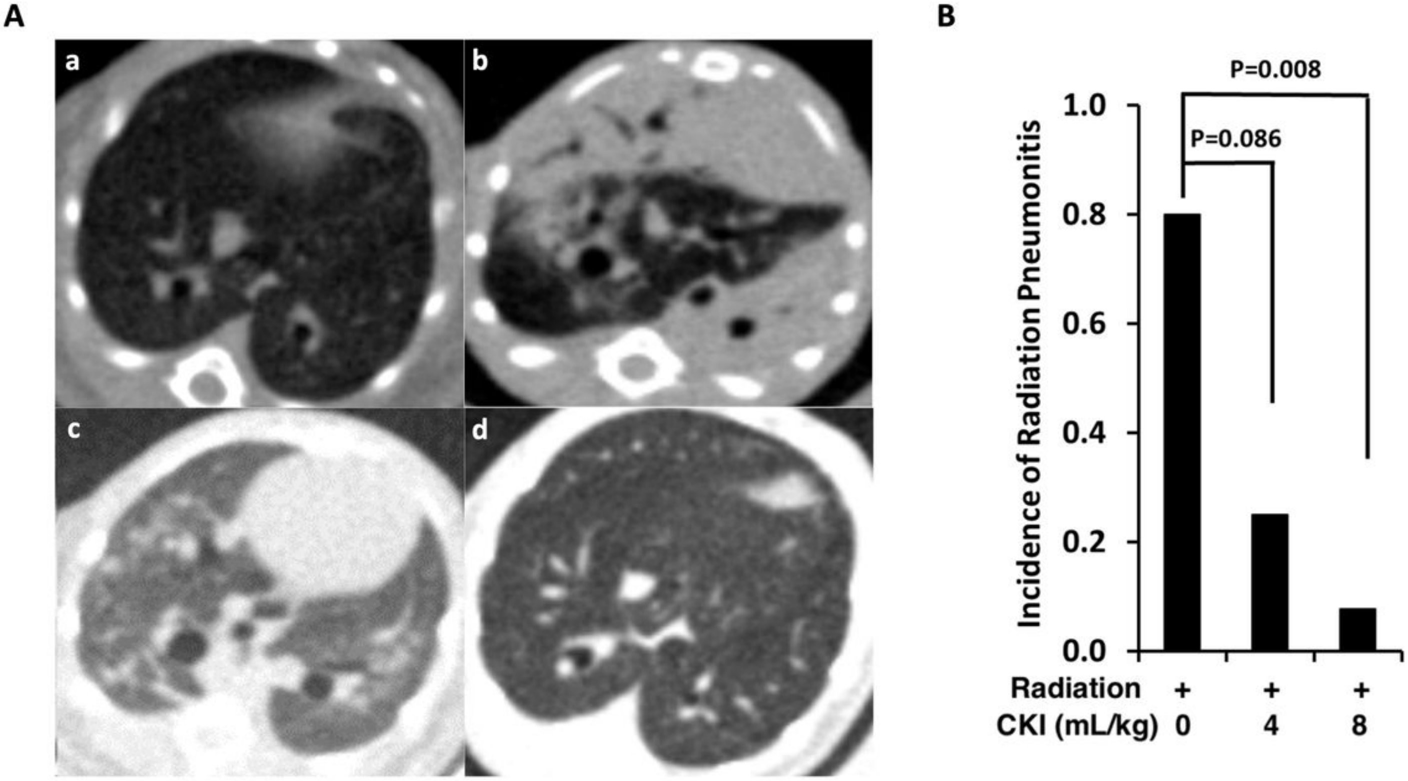

### Figure 3

**RILI incidence in TLI-treated C3H mice**. Micro-computed tomography (CT) imaging was conducted to estimate lung tissue damage and the protective effects of CKI treatment in mice 4 to 12 weeks after radiation of 13.5 Gy. (A) Representative micro-CT images of lung tissues from: (a) normal, nonirradiated mice; (b) mice treated with TLI + vehicle that developed RILI; (c) mice treated with TLI + 8 mL/kg CKI that developed RILI; and (d) mice treated with TLI + 8 mL/kg CKI that did not develop RILI. (B) Incidence of RILI in mice treated with TLI + vehicle control, TLI + 4 mL/kg, and 8 mL/kg CKI.

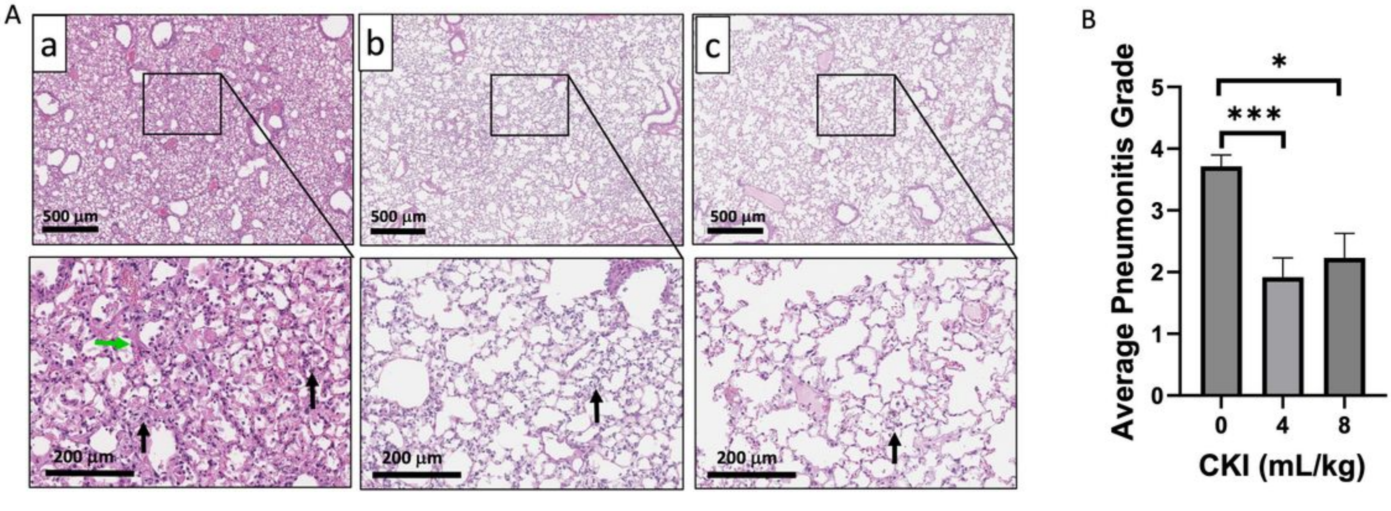

### Figure 4

**Histopathological analysis of lung tissue from TLI-treated mice.** (A) Images of hematoxylin and eosin stained lung tissues at 5x and 20x magnifications obtained from mice at termination of the study treated with: (a) TLI + vehicle control mouse showing lung with marked pneumonitis – marked interstitial inflammation (black arrow) and edema (eosinophilic proteinaceous exudate) filling alveolar spaces and thickening the alveolar walls (green arrow); (b) TLI + 4 mL/kg CKI mouse showing mild interstitial pneumonitis with mild inflammation (black arrow); (c) TLI + 8 mL/kg CKI mouse showing minimal interstitial pneumonitis with rare inflammatory cells infiltrated into alveoli (black arrow). (B) Mean pneumonitis grades in mice treated with TLI plus 0, 4, or 8 mL/kg CKI (n = 4-5 per group). Data are presented as the mean ± the standard error. **P*< 0.05.

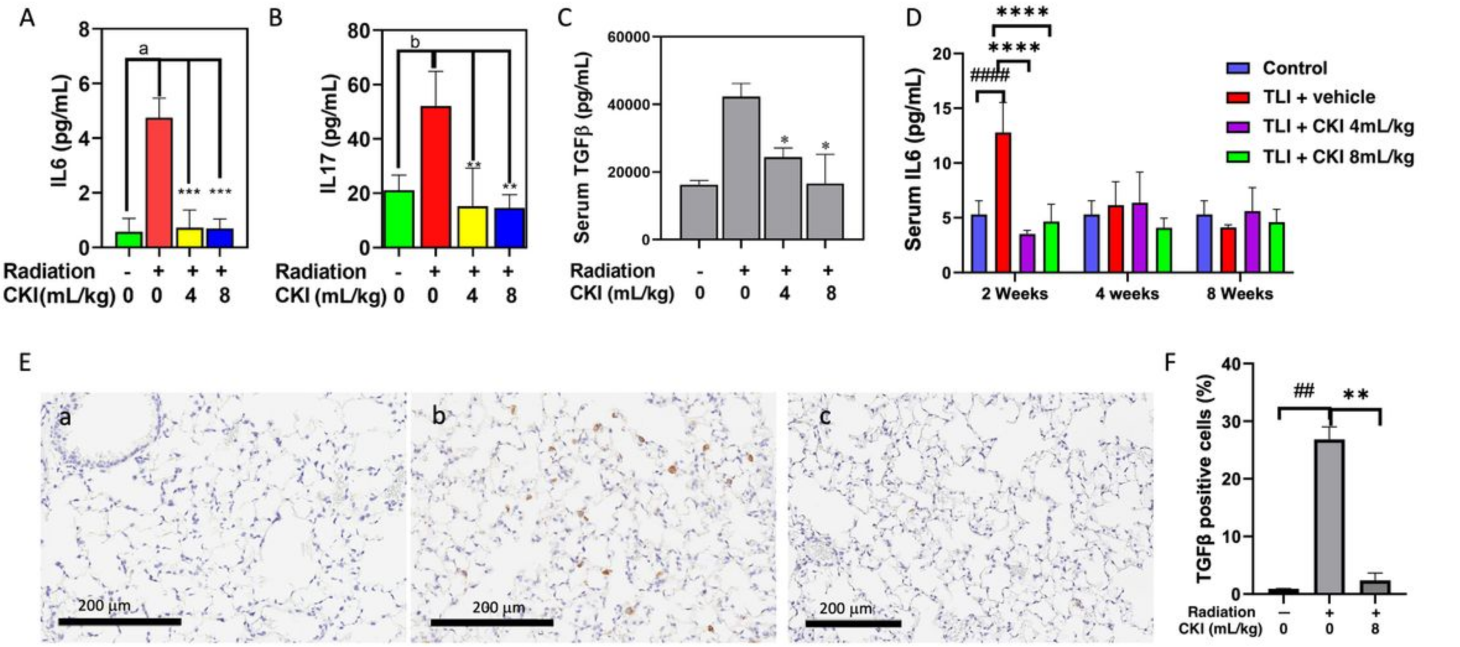

### Figure 5

**Proinflammatory cytokines in serum and lung tissues of C3H mice treated with TLI with or without CKI.** Serum cytokine levels of: (A) interleukin (IL) 6; (B) IL17A; and (C) transforming growth factor β (TGF-b) at 72 h after mice were exposed to 13.5 Gy radiation and treated with vehicle control or CKI (n = 3-4 per group). Measurements were made using ELISA. (D) Serum levels of IL6 at 2, 4 and 8 weeks after the mice were exposed to 13.5 Gy radiation and treated with vehicle control or CKI (n = 3-4 per group). (E) Immunohistochemistry analysis of TGF-β in lung tissues of C3H mice: (a) normal, (b) TLI (13.5 Gy), (c) TLI + CKI (8 mL/kg), at 2 weeks after radiation and CKI treatment. (F) Quantitative analysis of TGF-β–positive cells in lung tissues. Data are presented as the mean ± standard deviation. * *P* < 0.05; ** *P* < 0.01; *** *P* < 0.001; **** *P* < 0.0001 versus irradiated vehicle control. [a b] *P* < 0.05; ## *P* < 0.01; #### *P*< 0.001 versus normal non-irradiated control.

Figure 6

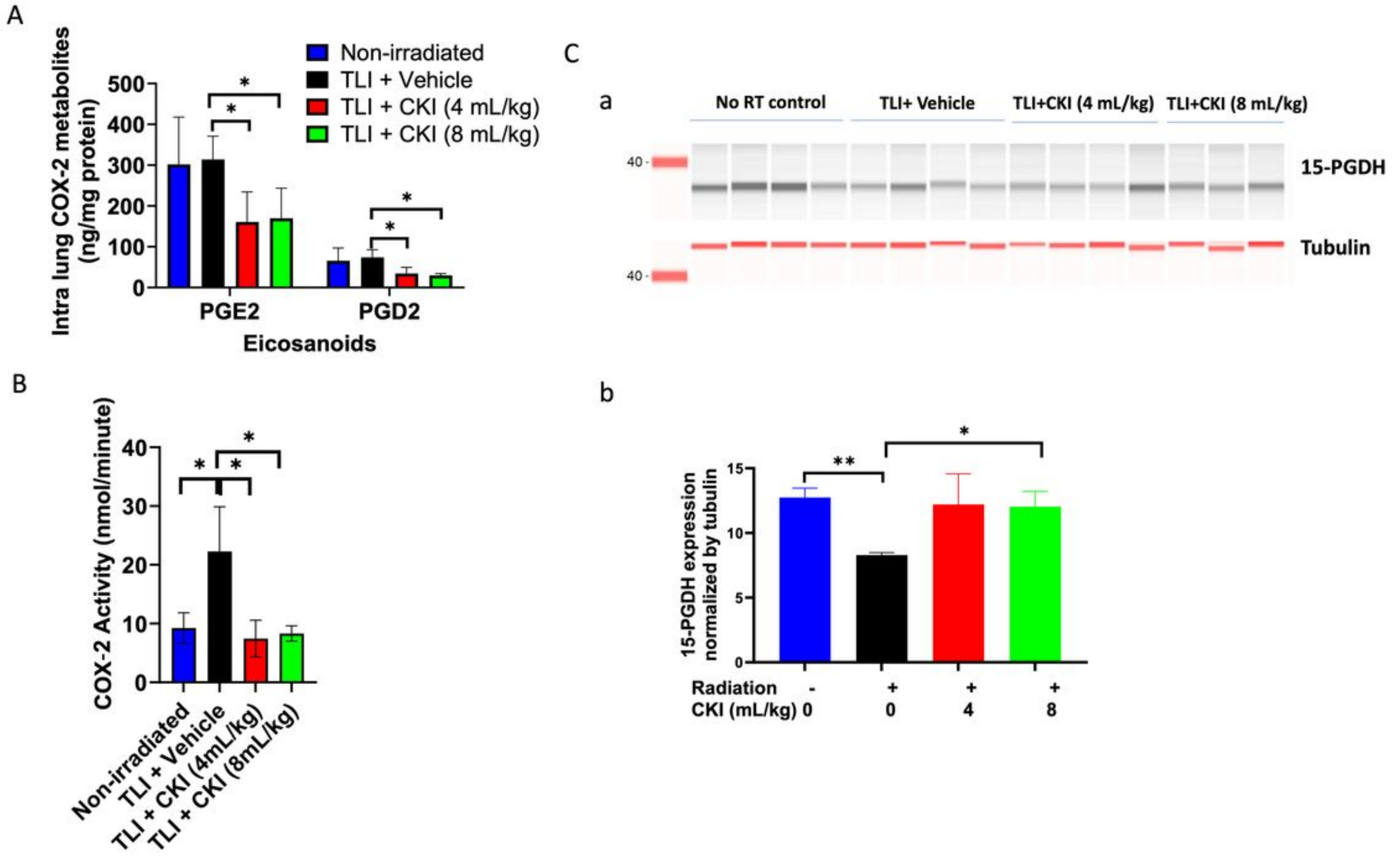

Figure 6

**The effect of CKI on COX-2 metabolic pathway. (**A) The intralung COX-2 metabolites $PGD_2$ and $PGE_2$ were measured using liquid chromatography/tandem mass spectrometry 4 weeks after irradiation in mice treated with TLI + vehicle and TLI + CKI (4 and 8 mL/kg). (B) The intralung COX-2 activity of C3H mice measured by ELISA kit. (C) Western blot analysis (a) and quantification of 15-PGDH protein expression (b) in C3H mouse lung tissues 4 weeks after irradiation and treatment with vehicle control or CKI. Data presented as mean ± SD. * $P < 0.05$; ** $P < 0.01$.

## Supplementary Files

This is a list of supplementary files associated with this preprint. Click to download.

- supplementarymaterialnew123023LC.docx
